# Cortical integration of tactile inputs distributed across timescales

**DOI:** 10.1162/IMAG.a.1146

**Published:** 2026-03-03

**Authors:** Wenyu Wan, K. Richard Ridderinkhof, Arko Ghosh

**Affiliations:** Cognitive Psychology Unit, Institute of Psychology, Leiden University, Leiden, The Netherlands; Department of Psychology, University of Amsterdam, Amsterdam, The Netherlands

**Keywords:** temporal integration, somatosensory, timescales, touch, ERP

## Abstract

Sensory experiences in the real world cut across timescales from milliseconds to seconds. Emerging evidence suggests that somatosensory processing is sensitive to the temporal structure of the stimuli in the sub-second scale, yet only a few select ranges within this scale have been studied. To process real-world information, the integration of tactile inputs must occur over a much broader temporal range. To address temporal integration across timescales, we studied scalp EEG signals from somatosensory cortex in response to a train of tactile stimuli presented to the fingertips with varying inter-stimulus intervals (ISIs) spanning hundreds of milliseconds to several seconds. We captured the variations in cortical signals as a function of the subsequent ISIs (next interval structure). We tracked cortical tactile processing through its early (<75 ms), intermediate (75 to 150 ms), and late stages (150 to 300 ms). We find that the early and late stages of cortical activity were sensitive to the previous ISI; EEG signals were suppressed with ISIs <500 ms and enhanced with longer ISIs, with this effect persisting even when ISIs were approximately 8 s. The intermediate stage of cortical activity was sensitive to both the previous and the penultimate ISIs. Our findings suggest that the specific somatosensory cortical processing stages integrate temporal structure across timescales to enable complex sensory experiences.

## 1 Introduction

The peripheral nervous system encodes texture by using the timing of tactile inputs in the millisecond scale (Weber et al., 2013). In the central nervous system, the cortex is capable of separating sequences of tactile information according to their temporal pattern in the scale of milliseconds to seconds (Bale et al., 2017). This capacity may serve diverse sensory functions—from localizing touch to learning behaviorally relevant patterns (Godde et al., 2000; Nguyen et al., 2016). In the real world, the temporal patterns cut across various time scales. For instance, the inter-touch interval distribution captured on the smartphone touchscreen is heavy tailed ranging from milliseconds to hours (Pfister & Ghosh, 2020). In the time scales spanning up to seconds, the interval dynamics may reflect the transient demands imposed by behavior. In these scales in particular, the somatosensory cortex may integrate rich tactile inputs according to the recent history of tactile events.

There is scattered evidence that the previous interval shapes somatosensory cortical processing, but the role of the penultimate interval is less clear. When the interval between the tactile stimulations is shortened, the somatosensory cortical response is attenuated (Espenhahn et al., 2020). A similar pattern of results is also found in studies using electrical stimulation of peripheral nerves (Kisley & Cornwell, 2006; Spooner et al., 2020), though this engages distinct sensors and pathways compared to the more naturalistic tactile stimulation (Pratt et al., 1979). Conventionally, the measurements are limited to few select intervals such as continuous tactile stimulations separated by 150 and 1050 ms in Espenhahn et al. (2020) and paired tactile stimulations separated by 30, 60, and 150 ms in Wuhle et al. (2011). For very short (<150 ms) intervals, the suppression may be under the influence of cortical excitability, with higher attenuation corresponding to lower excitability (Lenz et al., 2012; Schwartz & Shagass, 1964). By using MEG and electrical stimulation, the signal attenuation has been localized to the primary and secondary somatosensory cortex (Hamada et al., 2002). The primary somatosensory cortex shows attenuation for previous intervals up to 100 ms, whereas the secondary somatosensory cortex shows attenuated signals for intervals up to 500 ms. A similar pattern of results is found when using tactile stimulation, albeit the exploration only spanned intervals of 30 to 150 ms (Wuhle et al., 2011). A mismatch paradigm using tactile stimulations with intervals of 3 s implicates the secondary somatosensory cortex in building expectations based on the prior stimulations (Andersen & Lundqvist, 2019). These results suggest that the tactile information lingers longer in the intermediate stage of cortical processing than in the early stage. The sustained information processing of preceding stimuli in the intermediate stage may allow both the previous and penultimate inputs to shape the somatosensory processing of the current input, as well as its modulation by the length of the respective inter-stimulus intervals, as long as the total sequence occurs within 3 s. If and how the previous and penultimate tactile pulses at intervals spanning >3 s shape the cortical processing is not clear.

The conventional analytical framework—involving only a few select intervals—is limited in its ability to address the role of rich temporal statistics in cortical processing. Furthermore, this framework can only capture a simple relationship between the temporal statistics and brain responses (e.g., the shorter the interval, the higher the cortical signal attenuation). By contrast, the framework of joint-interval distribution (JID) allows us to separately measure tactile experiences according to their next-interval dynamics (i.e., the duration of the previous interval relative to the duration of the penultimate interval). This framework has been previously used to capture the rich temporal statistics of spiking neurons (Eagan & Partridge, 1989; Fitzurka & Tam, 1999) and smartphone touchscreen interactions (Ceolini & Ghosh, 2023; Ceolini et al., 2022; Duckrow et al., 2021). According to this framework, the stimulation triad can be separated according to the joint properties of the previous interval (say *k*) and the penultimate interval (say *k*-1), and then gathered in two-dimensional bins (b_n_ × b_n_ number of bins) spanning the JID. Here, an event-related potential (ERP) signal that reflects somatosensory processing can be estimated separately for each of the two-dimensional bins (henceforth called JIERP). This approach can capture more complex relationships than when using only few select intervals. For example, the ERP waveform associated with short consecutive intervals may be differently attenuated compared to the waveforms associated with short followed by long intervals. There is the possibility (although with little evidence) that the cortical activity reflects intervals prior to the penultimate interval (say *k*-2, *k*-3, *k-4*, so on) but such a feature space would require substantially more stimulations to uniformly cover all the additional dimensions than possible in a single laboratory session. Moreover, it will make interpretability more challenging. Finally, it is premature to add these extra intervals into the framework, given how little we know about the more recent and likely candidates: the previous (*k*) and the penultimate intervals (*k-1*).

The different stages of sensory cortical processing may be distinctly influenced by temporal statistics. This influence can be captured using the signal latencies of the ERPs derived from the separate two-dimensional bins of the JIERP. Therefore, this JIERP framework can in theory capture a rich variety of influences of temporal statistics on brain activity. To reveal these typical dynamics, the high-dimensional feature space (b_n_ × b_n_ bins of the JIERP × T signal processing latencies of the ERP, where b_n_ is the number of bins) can be reduced by using non-negative matrix factorization (NNMF; Lee & Seung, 1999). NNMF is a dimensionality reduction technique that can help reveal hidden patterns in the data by decomposing a matrix into lower-rank matrices (not unlike principal components, but resulting in additive rather than orthogonal components). The reduction can yield intuitively interpretable and low-dimensional prototypical patterns (so-called meta patterns). The interpretability stems from its parts-based decomposition, such as isolating the eyebrows of a face, and the additive nature of these components, where features like eyebrows, nose, and lips combine to reconstruct the full face (Lee & Seung, 1999). This method of reduction has been used to reduce a range of complex datasets from identifying facial features in images to human behavioural patterns captured on the smartphone (Ceolini & Ghosh, 2023; Lee & Seung, 1999). Here, the NNMF can help reduce the JIERP matrix into a set of prototypical *meta-JIERP* and *meta-time*. In essence, the *meta-JIERPs* represent how the cortical signals are modulated by preceding tactile pulses as a function of the penultimate and previous intervals, whereas the *meta-times* represent how the patterns of modulations are distributed over the different signal processing stages.

In this study, we studied the EEG signals evoked by tactile stimulations separated by a range of previous and penultimate intervals spanning 100 ms to 8 s. The tactile sensory density is high at the fingertips (Johansson & Vallbo, 1979), and the information from each tip is transmitted through a distinct set of nerve fibers to non-overlapping cortical representations (Sutherling et al., 1992). These differences guide us to test different fingers and analyse them separately. Furthermore, interleaving the stimulations within the same block may induce spatial integration, in particular surround inhibition (Biermann et al., 1998; Gandevia et al., 1983). Therefore, the stimulations were presented at the fingertips in dedicated blocks targeting right thumb, right index, or right little finger, and the brain responses were analysed independently for each finger as described below. We used the JID framework yielding 5 × 5 two-dimensional bins. The ERPs spanned an epoch from 200 ms prior to the tactile pulse to 299 ms following the pulse. This yielded a combined JIERP feature space of 5 × 5 × 500 two-dimensional bins. We reduced the JIERP after stimuli onset (5 × 5 × 299 two-dimensional bins) into interpretable prototypical patterns by using NNMF. To preview, these *meta-JIERPs* and *meta-times* revealed that the intermediate stage of cortical processing play a role that stands in contrast to the early or late stages. Here, we analyzed all the time points of the ERP but to communicate our results we use the terms early, intermediate, and late to refer to the different stages of sensory processing spanning the latencies of 1–75, 75–150, and 150–300 ms, respectively.

## 2 Methods

### 2.1 Participants

This study is part of a large data collection effort involving long-term smartphone behavioral logs and EEG in the laboratory. Participants were recruited from a pool accumulated through the agestudy.nl data collection platform. This platform leverages the Dutch brain registry (Zwan et al., 2021). Sixty-four healthy participants (age range: 20–81; 40 female) were enrolled. However, the self-reported health status of 4 participants altered leading to eliminations (one had tinnitus, one had stroke, one had arthrosis, and one had a psychiatric condition). All participants provided the informed consent, and the study was approved by the Ethics Committee of Psychology, Leiden University (ERB-reference number: 2020-02-14-Ghosh, dr. A.-V2-2044).

### 2.2 Tactile stimulation

We used tactile stimulations while recording the EEG signals (see below). A continuous train of tactile stimuli was applied to participants’ three fingertips (right thumb, right index finger, and right little finger) by using miniature electromagnetic solenoid-type stimulator (Dancer Design, North Yorkshire, England). The stimulator was controlled by using the parallel port of a computer and MATLAB (MathWorks, Natick, USA). Supra-threshold stimulations (lasting 10 ms) were delivered in blocks focused on a select fingertip. Each fingertip received ~200 stimulations per block, and nine blocks of stimulations were delivered (~600 stimulations over the recording each fingertip, ~1800 stimulations in total). The fingertip focus was randomly selected from one block to the next. The intervals between the stimulations were varied according to a random selection of intervals ranging from 100 to 10,000 ms. Participants were offered a short break every ~15 min and the entire stimulation session lasted for about 1 h. In order to cover any noise generated by the stimulators, we played a documentary series (David Attenborough’s Africa) on a screen and delivered the audio stream via a neck speaker. Furthermore, the movie allowed subjects to remain engaged through the long observation period, preventing boredom and sleep, and compliant with instructions aimed at minimizing movement artefacts: as summarized here (Vandewouw et al., 2021) and used here (Balerna & Ghosh, 2018). We note, apart from enabling a naturalistic state as opposed to instructed rest (Finn & Bandettini, 2021), movie-watching does not substantially influence the amplitude and adaptation of somatosensory-evoked potentials (Espenhahn et al., 2020).

### 2.3 EEG recordings and preprocessing

EEG data were collected by using 64 channels active EEG cap (62 scalp electrodes, 2 ocular electrodes; Easycap Gmbh, Wörthsee, Germany) with customized equidistant layout (Haenzi et al., 2014). The EEG signals were gathered referenced to the vertex (subsequently, the average reference was derived, as indicated below) and amplified with BrainAmp amplifier (Brain Products GmbH, Gilching, Germany). The signals were recorded and digitalized at the sampling rate of 1 kHz. The participants arrived for the measurement with washed hair and scalp, and we further degreased the skin at the contact sites by using alcohol swabs. We applied Supervisc gel (Easycap GmbH, Herrsching, Germany) to obtain an electrical contact between the skin and electrode, and targeted an impedance under 10 kΩ for each electrode. The continuous EEG recording was started prior to the tactile stimulations and stopped after the final stimulation.

**Fig. 1.**
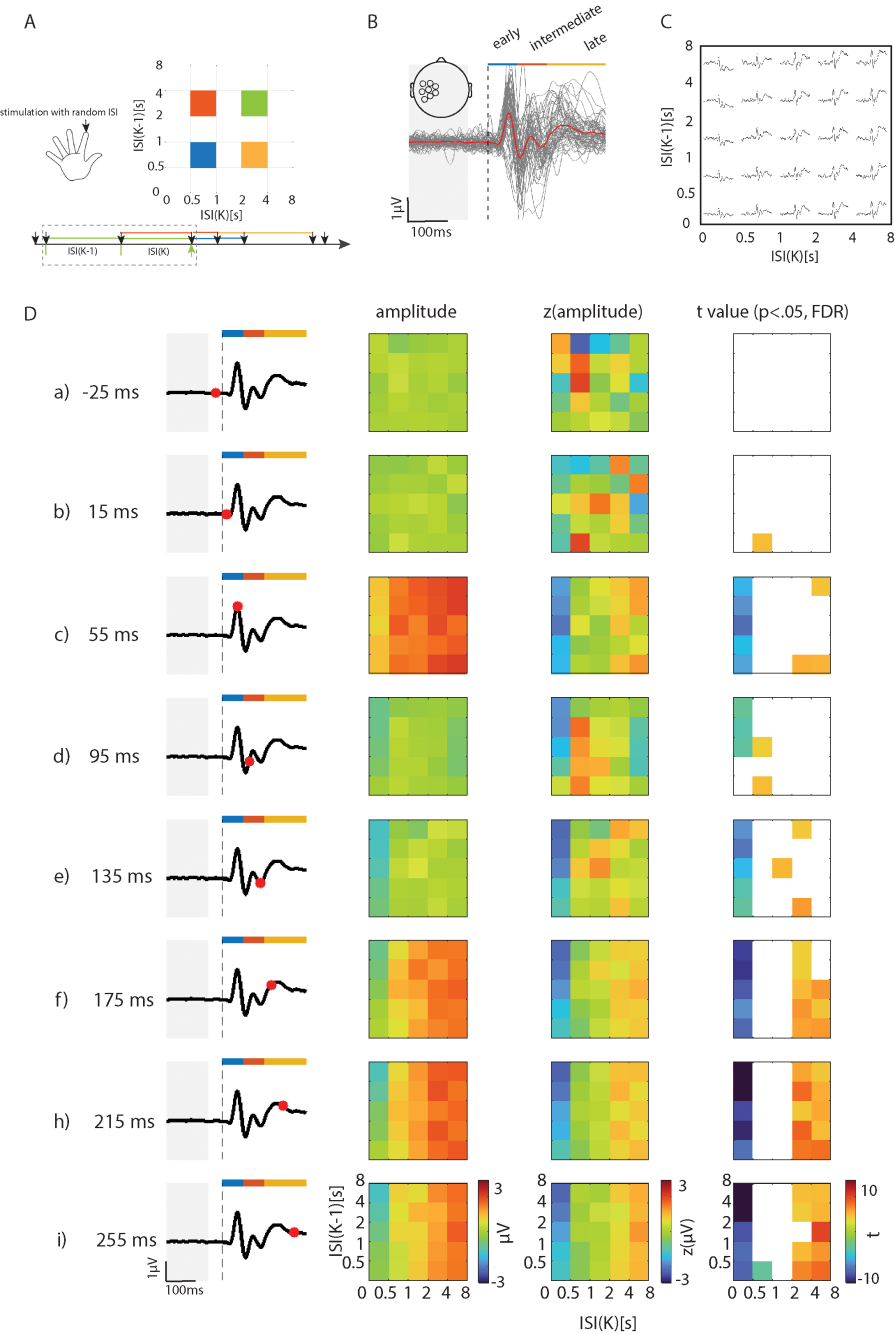
The temporal dynamics of tactile inputs shape cortical processing. (A) Schematic overview of the approach used to link the next-interval dynamics of tactile stimulations to cortical processing. A train of tactile stimuli with varying intervals (from 100 ms to 10 s) was applied to the index fingertip. The stimulations were separated according to the joint properties of the previous interval (*k*) and the penultimate interval (*k*-1) into 5 × 5 two-dimensional bins. For example, the last event (marked by the bottom green arrow) in the triad within the dashed rectangle belongs to a green bin in the upper JID space based on the previous interval (k) and the penultimate interval (k−1), both of which range from 2 to 4 s. (B) At the level of each individual, the electrode with the largest amplitude at short latencies was chosen from a pre-designed region of interest. The electrode locations of this region is shown as insert. The plot shows the grand-averaged tactile evoked potentials across the sample. The blue, red, and yellow bars marked the early (1–75 ms), intermediate (75–150 ms), and late stages (150–300 ms), respectively. The grand-averaged signal is shown using a red line and the evoked potentials from each individual are shown in grey. The grey contour marks the baseline to which individual ERPs were aligned. (C) An example of separating the ERPs according to the next-interval dynamics (the JIERP). (D) Population level JIERP based on the grand-average EEG signals. The first column shows the ERP signal disregarding the next-interval dynamics to help approximate the stage of sensory processing. The second column shows the JIERP of signal amplitudes at different latencies. The third column re-scaled the amplitudes (*z*-score) across the current timepoint. The fourth column reveals which two-dimensional bins were consistently suppressed or enhanced based on mass-univariate one-sample *t*-tests of the *z*-scored amplitudes (multiple comparisons was corrected by using false discovery rate (FDR) applied across the entire JIERP).

The data were processed using the EEGLAB toolbox (version 2021; Delorme & Makeig, 2004) on MATLAB 2022b (MATLAB, Mathworks, Natick). Any recorded electrode with impedance >10 kΩ was excluded. We removed ~4 electrodes for each participant on average. The data were band-pass filtered between 0.1 and 75 Hz. We performed independent component analysis (ICA), setting the number of components equal to the number of remaining electrodes. Eye-blink related EEG signal artefacts were subsequently removed based on the ICA decomposition (Pontifex et al., 2017). We removed ~1 independent component for each participant on average. The remaining independent components were back-projected using *pop_subcomp.mat* function of the EEGLAB toolbox. Next, the data were further band-pass filtered between 1 and 45 Hz using EEGLAB’s *pop_eegfiltnew* function with default settings (Delorme & Makeig, 2004). This function applies FIR filter method (Widmann et al., 2015). The full filter specifications are described in Supplementary Table S1. Excluded channels were subsequently interpolated using the spherical interpolation method (*pop_interp*). The continuous data were epoched around the stimuli using a temporal window of 500 ms, that is, - 200 to 299 ms. Any epochs with signals beyond ±80 μV were excluded from further analysis. Across the sample, the average number of trials remaining was 1667 (±170 SD). The data were re-referenced to the average value of the scalp electrodes. Each epoch was baseline corrected by subtracting the signal mean in the period -200 to -50 ms. Given the P50 as a typical component for somatosensory processing, the electrode with the largest short-latency signals (latency range of 25 to 75 ms) placed over the left somatosensory cortex (as Fig. 1B inserted showed) was chosen for each participant for further analysis. To capture the long-latency process, we focused on the electrodes around the vertex. The exact choice of the central electrode was based on maximum amplitude in the latency range of 150 to 300 ms (see Supplementary Fig. S1A).

### 2.4 JIERP

We adapted the joint-interval distribution (JID) approach to separate the tactile stimulations according to their next interval dynamics. We recently introduced this approach for the study of real-world behavioral event intervals (Ceolini & Ghosh, 2023; Duckrow et al., 2021), and here we introduced this approach for the study of passive tactile stimulations.

Here, a previous interval (say *k*) is described in conjunction with the penultimate interval (say *k*-1) as Figure 1A showed. The tactile joint intervals (based on the stimulation triads) were binned into 5 × 5 two-dimensional bins with edges (upper bound) of 0.5, 1, 2, 4, 8 s. Single-trial ERPs were estimated from the last event of the triad, and pooled together with other single-trial ERPs belonging to the same two-dimensional bin. Then, for each two-dimensional bin we estimated the tactile-related potential (ERP) by averaging the pooled signals. The threshold number of trials necessary to estimate the ERP was set to 10. Note, although participants were stimulated with ISIs up to 10 s, the next quadratic bin range, that is, 8 to 16 s, contained too few trials to be considered further. We obtained JIERPs for each individual. For grand-averaged JIERPs, we additionally averaged the JIERP across the individuals. Note, to reveal whether a particular bin’s amplitude was higher or lower than other bins at a given latency of the ERP, the grand-averaged JIERP was normalized by using *z*-score (across the bins) at each of the latencies.

### 2.5 Mass-univariate statistical analysis

To reveal which two-dimensional bins were consistently suppressed or facilitated across the JIERP, we used mass-univariate one-sample *t*-tests of the *z*-scored amplitudes at all the time points of the ERP. Multiple comparisons were corrected by using false discovery rate (FDR, using the function *fdr.mat* from eeglab toolbox, Delorme & Makeig, 2004) applied across the entire JIERP (*α* = 0.05).

To quantify the similarities of the JIERP at any given latency to the rest of the latencies, we used grand-averaged signals. This approach allowed us to focus on consistent signals present across individuals. A cross-correlation analysis was employed based on *z*-transformed grand-averaged JIERP using the Spearman method. Multiple comparisons were corrected by using the FDR method (*α* = 0.05). The values along the diagonal of the cross-correlation matrix (*ρ* = 1, window of 25 ms from the diagonal) was eliminated from the FDR to avoid overestimation of significance.

### 2.6 Interpretable data-driven dimensionality reduction

In the mass-univariate analysis above, each two-dimensional bin of the JIERP is considered independently, rather than considering the JIERP as a whole. Essentially, more complex patterns may exist which are hard to grasp using simple linear modelling or cross-correlations. For instance, there may be typical patterns of next-interval dependent signal modulations involving various two-dimensional bins but distributed across distinct latencies. To extract such typical patterns across latencies of the JIERP, we leveraged non-negative matrix factorization (NNMF). This method was originally demonstrated in facial image analysis, revealing key facial features (Lee & Seung, 1999), and extended to the study of behavioral JID to discover prototypical behaviours (Ceolini & Ghosh, 2023). Given NNMF can yield different factorization results due to its sensitivity to initial conditions (Wu et al., 2016), we applied a variant of the method known as stable and reproducible NNMF (starNNMF) described here (Ceolini & Ghosh, 2023). This method helps isolate the factorization that is most commonly visible across repetitions, that is, the most reproducible when run 1000 times. In brief, at the level of each individual, we applied the NNMF on a matrix of JIERP, where the EEG signal magnitudes (absolute value) were z-scored across bins for each latency. Because this method requires non-negative inputs, we subtracted minimum of the *z*-transformed matrix from the entire matrix. The subtraction using a common value preserves the relative signal features of the matrix. As shown in Figure 3A, the dimensional reduction method yielded a lower-rank approximation with prototypical patterns: how the signals were modulated across the two-dimensional bins (*meta-JIERPs*), and the corresponding latencies where the pattern was present (*meta-times*). We determined the optimal cluster number using cross-validation (with search ranging from 2 to 10 ranks) by calculating the error between the real matrix and the reconstructed matrix. The stable reduction was established based on 100 repetitions, isolating the commonly observed decompositions irrespective of the initialisation. We further clustered similar *meta-times* across the population by using *k*-means clustering (1,000 repetitions). The optimal number of clusters was determined using the *silhouette* method (*evalclusters*, MATLAB), which evaluates how well data points fit within clusters by maximizing separation and cohesion (Kaufman & Rousseeuw, 2009). The optimal cluster size search ranged from 3 to 7 clusters (range inspired by Haig et al., 1995; Mahini et al., 2023; Williams et al., 2015). Silhouette-based cluster diagnostic plots corresponding to the selected optimal number of clusters are shown in Supplementary Figure S11. For each cluster, we computed the average meta-time and its corresponding meta-JIERP by averaging all meta-times and meta-JIERPs assigned to that cluster, yielding a representative pattern.

At the level of population, we applied NNMF to address how the grand-averaged JIERP signals were modulated according to the next interval temporal structure of the stimuli. The NNMF was applied using the same steps as described above but the *k*-means clustering was not applicable in this case (as the grand-averaged JIERP resulted in only one set low rank approximations).

## 3 Results

### 3.1 Tactile-evoked potentials and the distinct stages of cortical processing

This report leveraged the high-dimensional JIERP approach to study cortical processing, but we shall first describe the simple event-related potential signal disregarding the next-interval dynamics to provide an overview of the signal features observed here. Note, throughout this section we focused on the findings from the stimulations presented at the index fingertip because it evoked the largest positive peak (with other stimulation sites described in the Supplementary Materials). In response to tactile pulse stimulation at the index fingertip, the evoked responses showed short-latency signals at the electrodes placed over the somatosensory cortex, while long-latency signals were observed over the central electrodes (Supplementary Movies 1 & 4). As expected, the short-latency signals from the electrodes located in somatosensory cortex showed components with peak latencies of ~50 ms (positive peak), ~75 ms (negative peak), ~100 ms (positive peak), ~150 ms (negative peak), and ~200 ms (positive peak). For subsequent analysis using the JIERP approach, given the P50 as a typical component for somatosensory processing, we focused on the electrode with the largest amplitude (latency search window of 25–75 ms) over the somatosensory cortex (Fig. 1B). In the remainder of the report, we used the terms *early*, *intermediate,* and *late* stages of the sensory processing to refer to the latencies of 1–75, 75–150, and 150–300 ms, respectively. Note, this labeling is only for convenient reading and all signal latencies were analysed. For the ERP stemming from the central electrodes, see Supplementary Figure S1. Across both the somatosensory and central electrodes, similar patterns as for the index finger stimulation were also captured in response to stimulation of the thumb and little finger (Supplementary Figs. S2, S3, S4, S5).

### 3.2 Mass-univariate analysis reveals the influence of recent temporal statistics on tactile-evoked potentials

To address how the tactile temporal statistics shapes the EEG signals, we separated the tactile ERPs according to the next-interval dynamics preceding the target stimulation (JIERP). Here, a matrix of ERPs was separated in two-dimensional bins, binned based on the previous and the penultimate intervals (Fig. 1C). We analyzed the differences in the ERP signal amplitudes across bins for all the latencies of the ERP (JIERP). At each latency, the amplitudes were *z*-scored across the bins, and one-sample *t*-tests (*z*-scores vs. 0) revealed the two-dimensional bins with distinctly higher or lower activations (Fig. 1D). Note, in this approach the bins were considered independently of each other (mass-univariate statistics). During the time window of [-50, 0] ms—after the ERP baseline period and before stimulus onset—there was no significant modulation in any given bin compared to the rest of the bins (p > .05, FDR-corrected). The differences became evident at the early stage (~50 ms post stimulation), such that the bins with short previous intervals <500 ms showed attenuated signals compared to the rest of the bins (p < .05, FDR-corrected). The attenuation appeared irrespective of the penultimate interval. At this stage, there was evidence for signal enhancement for bins composed of short (<500 ms) penultimate intervals followed by long (>2,000 ms) intervals (p < .05, FDR-corrected). The subsequent stage of processing, the intermediate stage, showed a more dynamic pattern of signal modulations which altered from one ERP peak to another. At the late stage of processing, bins with previous intervals <500 ms showed attenuated signals compared to the mean across bins (p < .05, FDR-corrected). Moreover, there was an enhancement of the signals with previous intervals >2,000 ms compared to the mean (p < .05, FDR-corrected). Consistent signal modulations in the JIERP were also observed when the thumb or the little finger was stimulated (Supplementary Figs. S2 & S4, Supplementary Movies 2 & 3).

We also analyzed the ERPs over the central electrodes, consisting of a broad signal peaking at ~ 200 ms. This entire signal was attenuated at the two-dimensional bins with previous interval <500 ms (p < .05, FDR-corrected). This pattern was observed for all three stimulation locations (Supplementary Movies 4, 5 & 6, Supplementary Figs. S1, S3 & S5).

### 3.3 Patterns of JIERP modulation at the early and late stages of somatosensory processing

**Fig. 2.**
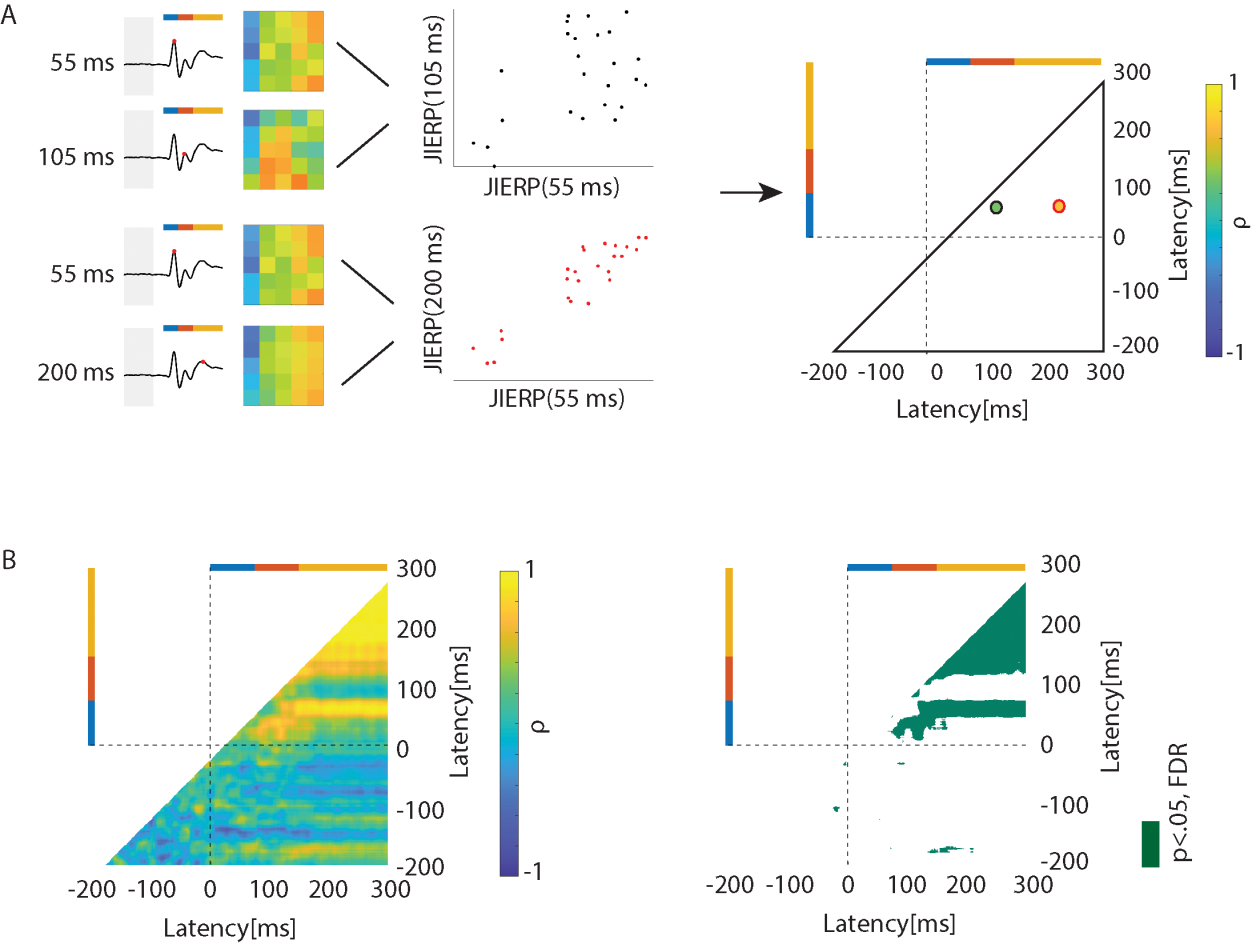
The JIERP pattern is shaped by temporal dynamics at distinct stages. (A) Schematic overview of the cross-correlation analysis for calculating the similarity of each time point of JIERP. Bar colors are identical to Figure 1. (B) Cross-correlation analysis results of tactile evoked JIERP of the index finger. The left panel is the coefficient plot, and the right panel is the p statistic plot (p < .05, FDR).

The JIERP revealed modulation of the EEG signals according to the two-dimensional bins based on the previous and penultimate intervals. As described above, the modulation varied according to the stage of processing for the somatosensory signals. We quantified the similarities of the JIERP at any given latency to the rest of the latencies by using correlations (Fig. 2A). For the index finger stimulations, cross-correlation analysis revealed statistically significant similarities (p < .05, FDR-corrected) with correlation coefficients (*ρ*) exceeding 0.5, indicating reasonably strong relationships between the JIERP patterns at the early and late stages. In contrast, the correlation between intermediate stage with the other processing stages was relatively small and not statistically significant (p > .05, FDR-corrected, see Fig. 2B). For the signals captured at central electrodes, the JIERP patterns showed similar modulations across the intermediate and late stages (Supplementary Figs. S3C & S5C).

### 3.4 Dimensionality reduction: Interval patterns relevant for somatosensory processing across the different stages of processing

**Fig. 3.**
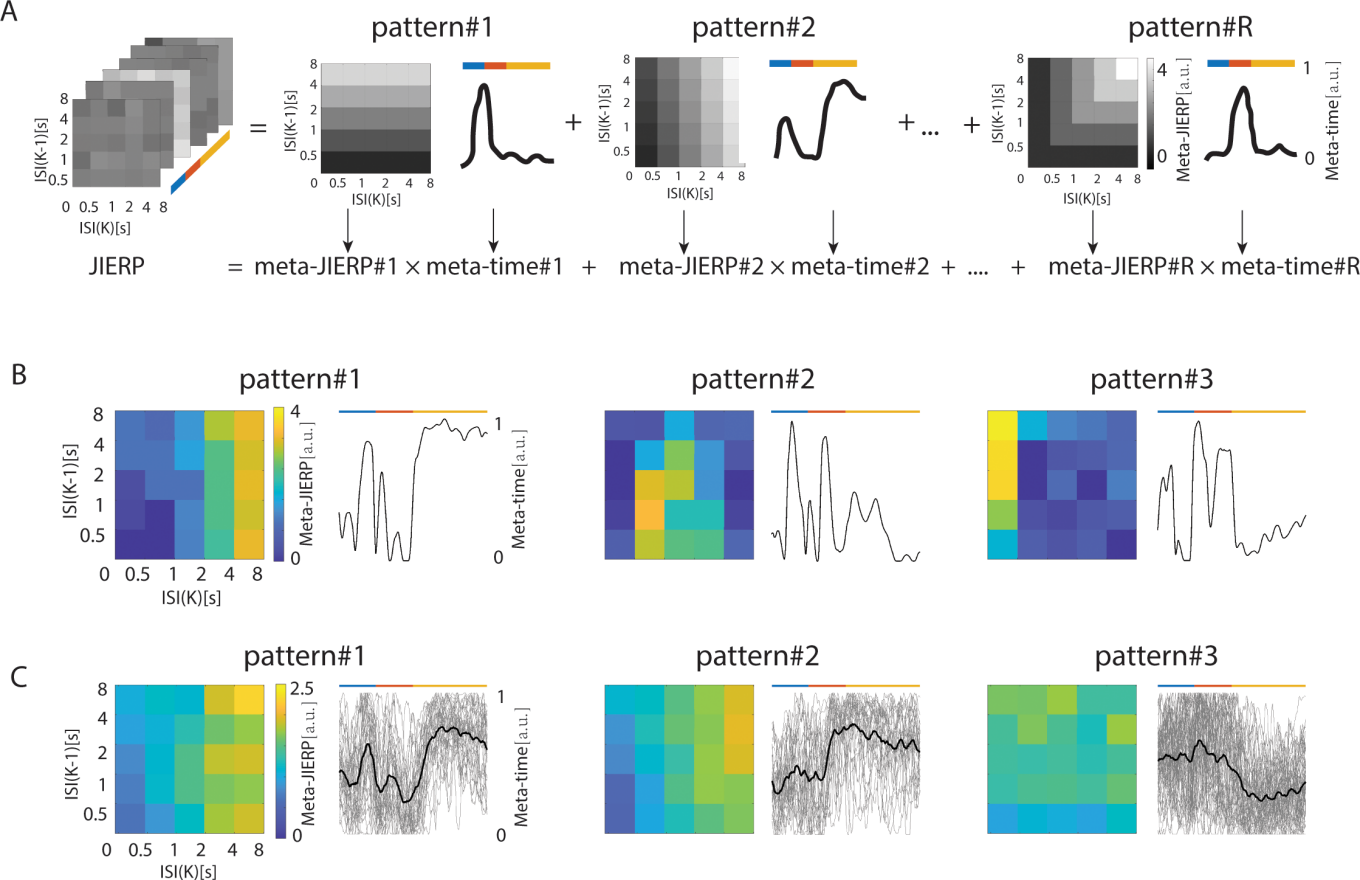
The typical JIERP patterns across different stages. (A) A sketch of the NNMF dimensionality reduction method. This method can yield low-dimensional and interpretable prototypical patterns (#1 to #R), which can indicate what the pattern is (*meta-JIERPs*) and when the pattern appeared (*meta-times*). (B) Three typical JIERP patterns at the population level, derived from the grand averaged JIERP. (C) same as (B) but showing three typical JIERP patterns at the individual-participant level. We obtained the typical JIERP pattern for each participant and then clustered *meta-JIERPs* using *k*-means (see section 2). Finally, we averaged *meta-JIERPs* and *meta-times* for each cluster. Note that a different scale is used here compared to (B). The averaged *meta-JIERPs* is depicted by a black line, while the *meta-JIERPs* for each participant is shown in grey. The corresponding statistical results are presented in Supplementary Figure S8A.

By using simple analytical frameworks, that is, mass-univariate statistics and correlation analysis, we established the dominant patterns underlying the JIERP, but these methods are not designed to extract time-variant patterns. Therefore, we next used data-driven dimensionality reduction to extract the key patterns of the rich JIERP matrix. Essentially, the JIERP magnitudes (z(absolute(JIERP)) – min(z(absolute(JIERP))), see section 2) spanning all the latencies were reduced in terms of a few *meta-JIERPs*, that is, prototypical interval-based modulations of the two-dimensional bins, and the corresponding *meta-times*, that is, the extent to which prototypical patterns were present at any given latency (Fig. 3A). To illustrate how the identified patterns are expressed at different analytical levels, we applied this analysis at the population level using the grand-averaged JIERP signal and at the individual level using the individual JIERP traces, and further examined these patterns in signals from the somatosensory and the central electrodes. Here, we focus on the results from the index finger (for the thumb and little finger please see Supplementary Figs. S6 & S7).

For the grand-averaged signal from the somatosensory electrodes, the dimensionality reduction revealed three prototypical patterns. We observed a prototypical pattern #1 (Fig. 3B) with the reduced matrix involving small values for short (<1 s) previous intervals, and large values for longer (>2 s) previous intervals (for follow-up statistics on this pattern, see Supplementary Fig. S8A). The corresponding meta-time showed large values at the early and late stages of processing. Pattern #2 involved large values for intermediate (>0.5 and <4 s) previous intervals (see Supplementary Fig. S8A). The corresponding meta-time peaked at early stages of processing with the second peak during the intermediary stage. Pattern #3 involved large values for short (<0.5 s) previous intervals (see Supplementary Fig. S8A). The corresponding meta-time peaked at the intermediary stage of processing.

We used the same approach at the level of each individual followed by a k-means clustering on the reduced meta-time. The corresponding meta-JIERPs were averaged across the clusters. This analysis yielded three prototypical patterns. We observed a prototypical pattern #1 (Fig. 3C) with the averaged meta-JIERPs involving small values for short (<1 s) previous intervals, and large values for longer (>2 s) previous intervals (for follow-up statistics on this pattern, see Supplementary Fig. S8A). The corresponding meta-time showed large values at the early and late stages of processing. Pattern #2 involved small values for short (<1 s) previous intervals, and large values for longer (>2 s) previous intervals. The meta-time of Pattern #2 showed large values at the later stages of processing.

For the same analysis but now on the central electrode signals, see Supplementary Figures S7 and S8B.

## 4 Discussion

We analyzed cortical signals in response to a train of tactile inputs on the right hand, with the interval between stimulation spanning hundreds of milliseconds to several seconds. Signal processing in the left somatosensory cortex was shaped by both the penultimate and previous intervals of tactile stimulation, and this influence was modulated by the respective inter-stimulus intervals spanning up to 8 s. The intervals modulated the cortical processing by using rules which varied according to the stages of processing. By using a series of analytical approaches, we showed that the early and the late stages of cortical processing were modulated by the interval patterns. In both of these stages, the signals were smaller when the previous intervals were shorter than 500 ms. The influence of the temporal statistics at the intermediate stage was modulated by a combination of the penultimate and previous intervals. Our findings indicate that our brain has the intrinsic ability to integrate tactile information according to temporal structures across the scales of milliseconds to seconds.

One of the prominent patterns we observed included small cortical signals detected at the early stages of processing for previous intervals <500 ms (Figs. 1D and 3B). This is consistent with prior studies (Hamada et al., 2002; Zhu et al., 2007). For instance, inter-touch intervals separated by 2 s resulted in larger amplitude in early stage of the ERP (<75 ms) compared to intervals separated by <500 ms; the late-stage ERPs (75–150 ms) were virtually abolished by the shorter intervals (Zhu et al., 2007). One possibility is that this pattern stems from a form of inhibition induced when processing the first input and reflected on the second input, akin to the paired-pulse inhibition observed in rodents (David-Jurgens & Dinse, 2010). Functionally, the small signals may allow neural populations to extract spatio-temporal information, as suggested by applying information theory on single neuron recordings in rodents (Liu et al., 2017). In our observations, the penultimate intervals played a role in shaping the signals. The fluctuations in the temporal matrix (meta-time) may be explained by a form of rebound activation, as often seen post-inhibition (Qi et al., 2016). Finally, there may be a concern that the signal fluctuations observed does not reflect altered neural activity but rather how the EEG signal evoked by the current stimuli summates with the previous stimuli. However, this is unlikely as we saw no signal changes in the segment that appeared after the baseline period and up to ~20 ms after stimuli onset. Furthermore, the random distribution of intervals within each bin minimized any systematic time-locked summation artifacts.

Another pattern of modulation consisted of amplitude modulations across the ‘diagonal’ of the joint-interval distribution: with small signals for consecutive short-short intervals and large signals for consecutive long-long intervals. The trend spanned the entire ~100 ms to 8 s range. We speculate that this pattern reflects mechanisms that help sustain neural representations toward temporal integration of event series, such as short-term memory or perceptual priming (Bermudez Contreras et al., 2013; Han et al., 2008). Animal studies suggest that this kind of reverberation of brain activity for past events can last up to a few minutes (Bermudez Contreras et al., 2013). Based on the ERP signal latencies, we suggest that the activity in the early stage is echoed or recurred in the late stage, resulting in the similarity of prototypical pattern between those two stages (Auksztulewicz et al., 2012; de Lafuente & Romo, 2006). Alternatively, top-down modulation at the sensory cortex (Ruff, 2013) may exist in the late-stage processing to get ready for the arriving stimuli.

Our results also indicated temporal integration at the intermediate stage of somatosensory processing. According to the dimensionality reduction revealed prototypical patterns corresponding to the intermediate stage. Our results are in line with previous studies indicating that complex temporal integration follows early somatosensory cortical processing (Rossi-Pool et al., 2021). However, exactly which mechanisms underscore these patterns remains unclear. It is possible that stimulation-induced inhibition, sustained neural representation, and links to extra-somatosensory processes (such as for motor control) all contribute to the distinct processing at the intermediate stage (Forss & Jousmaki, 1998; Haggard & Whitford, 2004).

In sum, the patterns captured in the so-called JIERP suggest that tactile processing is shaped according to the recent temporal context. This parallels the emerging view from both human and animal studies that cortical sensory processing is state-dependent (Parajuli et al., 2023; Russell et al., 2024; Taga et al., 2018; Zagha et al., 2013). In rodents, there is rapid adaptation in somatosensory cortical neurons in the form of reduced amplitudes for the subsequent stimuli in a train of stimulation separated by fixed intervals (Chung et al., 2002). However, this adaptation is prominent in quiescent but not in a behaviorally engaged state (Castro-Alamancos, 2004). Whether our observations stem from bottom-up (i.e., tactile stimuli driven) or top-down (i.e., behavioral or background states) processing was unclear. To elaborate, our data were collected during movie viewing—which evokes a rich array of background states as opposed to the simplistic states observed during traditional rest (Meer et al., 2020). Our tactile stimulations spanned across these putatively diverse states and the JIERP was constructed by averaging signals across trials for each bin, arguably averaging out the influence of the background brain states driven by movie viewing. According to this perspective, the patterns observed here are more likely to be modulated by bottom-up process than top-down states. But this also raises a new branch of questions: if the temporal context drives the brain into different states to ultimately influence tactile processing, how is this achieved? For instance, there may be rather complex cascades that shape the tactile processing. Say short consecutive tactile stimulations can increase the motor excitability to ultimately activate the same circuits also responsible for attenuating self-generated tactile inputs (Bays et al., 2006; Ridding et al., 2000; Sakamoto et al., 1987). Addressing such processes requires the development of analytical tools building on the JIERP so that one can study such cascades and separate the background state changes from states induced by touch trains.

One consequence of the JIERP is that each two-dimensional bin consists of only a few trials, even in an hour-long recording session. While ERP signals can be derived from as few as six trials (Boudewyn et al., 2018), EEG signal processing to remove blink or muscular artifacts, and conditions such as electrical insulation to avoid measurement noise may be necessary prerequisites for this approach. It remains to be addressed if our approach can be used in less controlled EEG recordings with higher measurement noise.

Our study provides a new perspective on studying how brain processes continuous sensory stimuli. Our findings raise the possibility that the intermediate stage of cortical processing may leverage the timing of previous and penultimate inputs to recognize the current state. Such a process may work in tandem with other feed-forward processes in early sensory processing to prioritize select input features (Westerberg et al., 2023). The putative processes may be widespread as similar pattern of results were observed for sensory stimuli applied to the thumb, index and little fingertip—and these locations are served by distinct cortical representations, peripheral nerves, nerve receptor density, and exposed to distinct sensory statistics in behaviour. Notably, there is an emergent understanding of the complex temporal statistics experienced in the real world where consecutive tactile event intervals can show large variations in the range of what is explored here (Pfister & Ghosh, 2020; Sundaram et al., 2019). The rise of smartphone use and its in-built sensors makes this vivid (Ceolini & Ghosh, 2023; Ceolini et al., 2022); touchscreen interactions can range from consecutive short intervals to long followed by short intervals when transitioning between apps. Thus, our study helps bridge the understanding of cortical somatosensory processing to the complex temporal patterns experienced in real-world behaviour. The JIERP approach introduced here may be extended to other body parts or even other sensory modalities to reveal the shared mechanisms underlying the processing of complex temporal patterns.

## Supplementary Material

Supplementary Material

Supplementary Movie 1

Supplementary Movie 2

Supplementary Movie 3

Supplementary Movie 4

Supplementary Movie 5

Supplementary Movie 6

## Data Availability

Pseudo-anonymized MATLAB.mat file containing processed JIERP data is made available on dataverse.nl upon publication; The custom-written scripts used towards this report are shared on: https://github.com/CODELABCODELIB/JID_ERP_Tactile_2024
