# Supplementary material for: Cortical integration of tactile inputs distributed across timescales
Source: Imaging Neurosci (Camb). 2026 Mar 3;4:IMAG.a.1146. doi: 10.1162/IMAG.a.1146 (PMC12961305; doi:10.1162/IMAG.a.1146)
Supplement: Supplementary Material [file IMAG.a.1146_supp.pdf]

Supplementary table 1. Specifications of the band-pass filter applied during EEG preprocessing.

Parameter	Value/Description
Type of filter	Hamming-windowed sinc FIR band-pass filter
Cut-off frequencies	Passband: 1-45Hz (-6 dB cutoffs at 0.5 and 45.5 Hz)
Filter order (length)	3300 (filter length=3301)
Transition bandwidth	~1Hz (EEGLAB default)
Pass-band ripple	Determined by Hamming window (not explicitly specified by EEGLAB)
Stop-band attenuation	Determined by Hamming window (EEGLAB default)
Filter delay and causality	Linear-phase FIR; Zero-phase / non-causal
Direction of computation	Two-pass / delay-compensated (EEGLAB default)
Sampling rate	1000 Hz
Nyquist & roll-off consideration	Nyquist = 500 Hz; low-pass cutoff (45 Hz) well below Nyquist, allowing adequate transition bandwidth

Supplementary methods

To examine whether a given bin significantly differed from the rest of bins, we conducted one sample Wilcoxon signed-rank tests comparing that bin to the remaining bins, excluding its neighboring bins to avoid spatial bias. Additionally, to assess potential gradients along a row (previous ISIs), column (penultimate ISIs), or diagonal (both previous and penultimate ISIs), we applied Kendall's tau correlation test between the bin values and their spatial positions.

Supplementary results

For the grand-averaged signal from the somatosensory electrodes, the dimensionality reduction revealed three prototypical patterns. First, we observed a prototypical pattern where short penultimate followed by short previous intervals showed attenuated signals and the signals were enhanced for longer previous intervals (supplementary Fig. S8A pattern #1, $p<0.05$, FDR-corrected). This trend was most evident along the diagonal of the meta-JIERP space, where shorter consecutive intervals were associated with greater attenuation (Kendall's τ =1, p = .0167). Second, we observed a prototypical pattern where previous intervals between 500 and 4,000 ms showed the larger values (supplementary Fig. S8A pattern #1, $p<0.05$, FDR-corrected). Third, we observed a pattern where previous intervals <500 ms showed larger values than longer previous intervals (supplementary Fig. S8A pattern #3, $p<0.05$, FDR-corrected), and within this range, the longer penultimate intervals showed a trend toward larger values (Kendall's τ =1, p = .0167).

We performed a similar analysis at the level of each individual and then clustered the results based on the *meta-times*. The corresponding *meta-JIERPs* were averaged across the clusters. This approach revealed three prototypical patterns. First, patterns #1 & #2 (supplementary Fig. S8A) showed the signals were attenuated for short consecutive intervals and enhanced for long intervals ($p<0.05$, FDR-corrected). The patterns were present in the early and the late stages of somatosensory processing (Fig. 3C pattern #1 & #2). Next, there were *meta-times* which indicated at the intermediate stage of processing with an enhancement for long (>2 s) penultimate intervals and an attenuation for short (<0.5 s) penultimate intervals ($p<0.05$, FDR-corrected).

For the same analysis but on the central electrode signals, see Supplementary Fig. S8B. For the same analysis on the other fingers, see Supplementary Figs. S9 & S10.

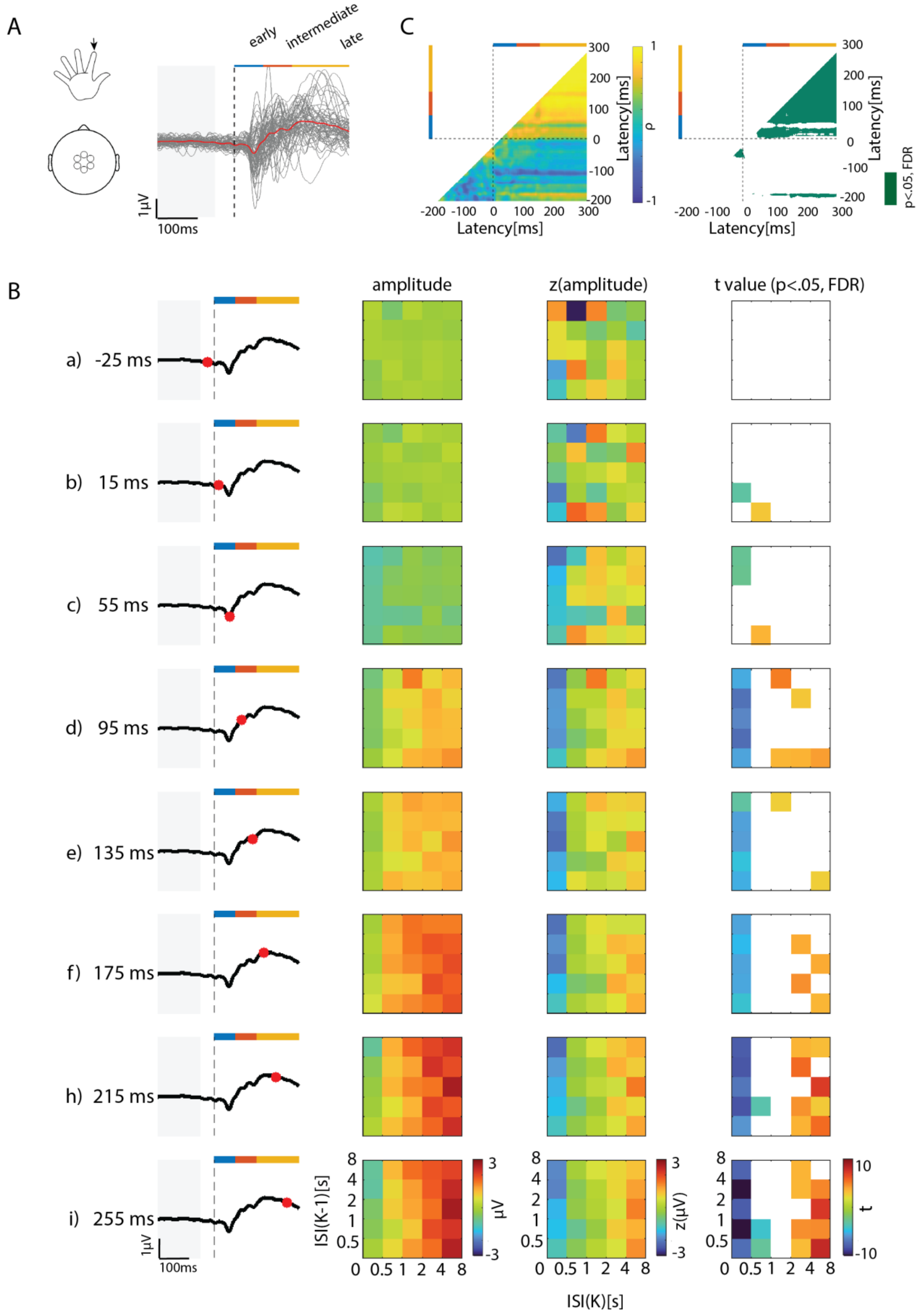
A
early
intermediate
late
1μV
100ms
C
Latency[ms]
ρ
p<.05, FDR
B
amplitude
z(amplitude)
t value (p<.05, FDR)
a) -25 ms
b) 15 ms
c) 55 ms
d) 95 ms
e) 135 ms
f) 175 ms
h) 215 ms
i) 255 ms
ISI(K-1)[s]
μV
z(μV)
t
ISI(K)[s]

Supplementary Fig. S1. The temporal dynamics of tactile inputs from index fingertip can shape the cortical processing. (A) At the level of each individual, the electrode with the largest amplitude at long latencies (150-300ms) was chosen. The central electrode locations were shown as insert. The grand averaged tactile evoked potentials across the sample were shown using a red line and the evoked potentials from each individual were shown in grey. Blue bar marked the early stage (from 1-75ms), red bar marked the intermediate stage (from 75-150ms), and yellow bar marked the late stage (from 150-300ms). (B) Population level JID-ERP based on the grand average EEG signals. The first column showed the ERP signal disregarding the next interval dynamics to help approximate the stage of sensory processing. The second column showed JID-ERP signal amplitudes at different latencies. The third column re-scaled the amplitudes (z-score) across the current ERP temporal latency. The fourth column revealed which two-dimensional bins were consistently suppressed or enhanced based on mass-univariate one-sample t-tests of the z-scored amplitudes (multiple comparisons was corrected by using false discovery rate, FDR applied across the entire JID-ERP). (C) Cross-correlation analysis results of tactile evoked JID-ERP of index finger. The left panel was the coefficient plot, and the right panel was the p statistic plot (survived by $p<.05$ and FDR).

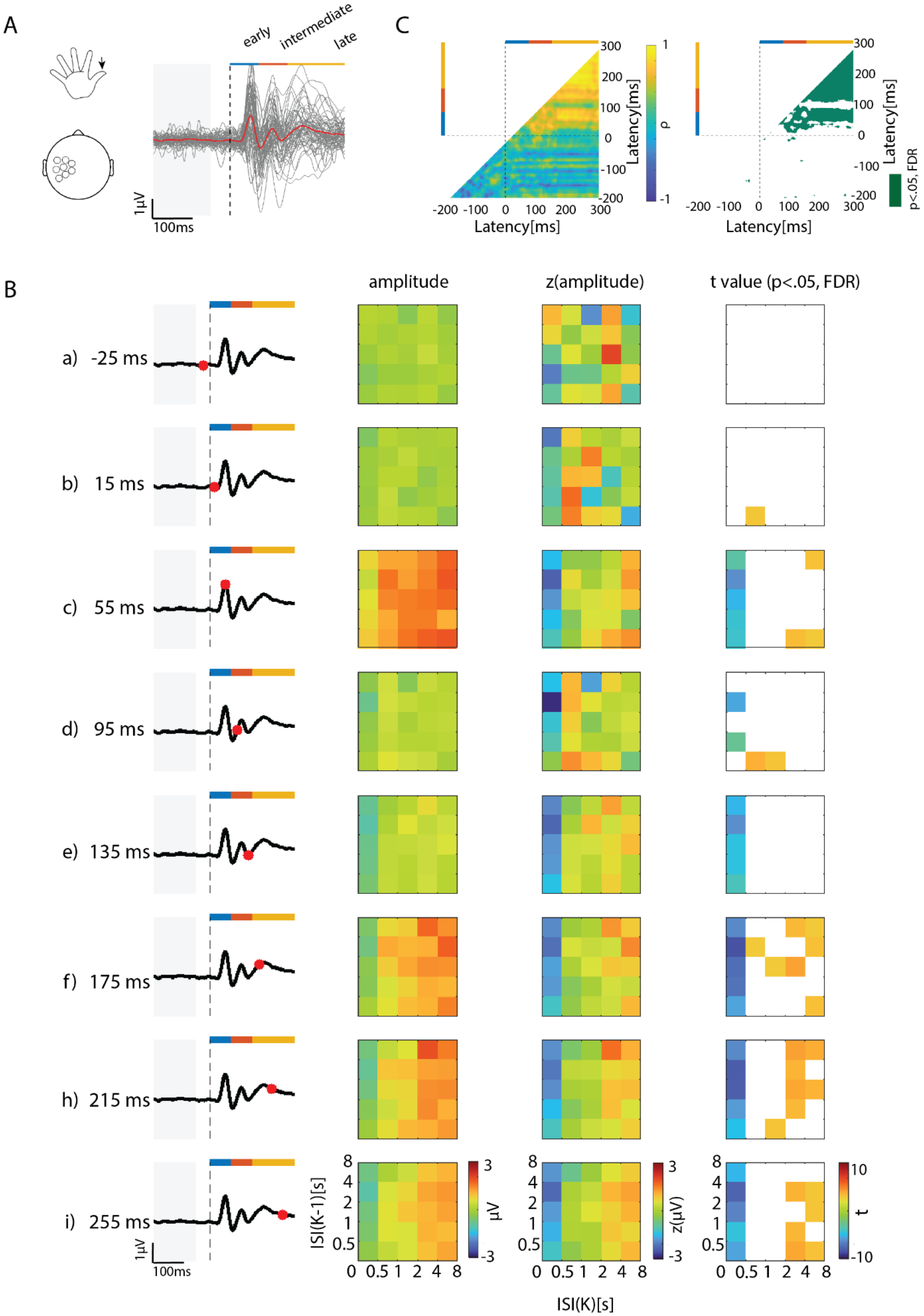

Supplementary Fig. S2. The temporal dynamics of tactile inputs from thumb fingertip can shape the cortical processing. (A) At the level of each individual, the electrode with the largest amplitude

at short latencies (25-75ms) was chosen. The electrode location placed over left somatosensory cortex is shown as insert. The grand averaged tactile evoked potentials across the sample. Blue bar marked the early stage (<75ms), red bar marked the intermediate stage (from 75-150ms), and yellow bar marked the late stage (from 150-300ms). The grand averaged potentials were shown using a red line and the potentials from each individual were shown by grey lines. (B) (C) Please see the supplementary Fig. S1 for legend.

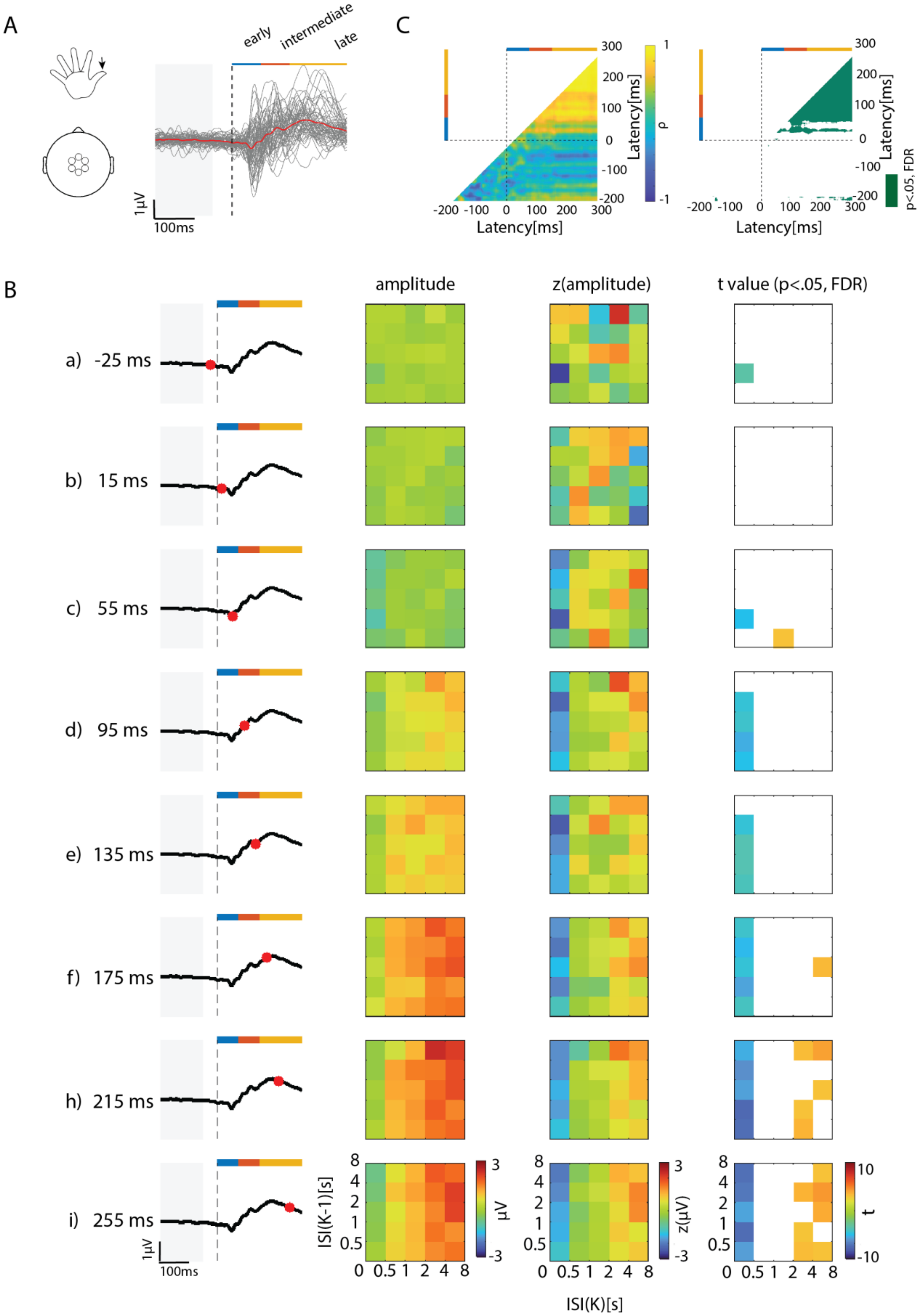
A
early
intermediate
late
1µV
100ms
C
Latency[ms]
ρ
p<.05, FDR
B
amplitude
z(amplitude)
t value (p<.05, FDR)
a) -25 ms
b) 15 ms
c) 55 ms
d) 95 ms
e) 135 ms
f) 175 ms
h) 215 ms
i) 255 ms
ISI(K-1)[s]
µV
z(µV)
t
ISI(K)[s]

Supplementary Fig. S3. The temporal dynamics of tactile inputs from thumb fingertip can shape the cortical processing at the central electrode location. Please see the supplementary Fig. S1 for legend.

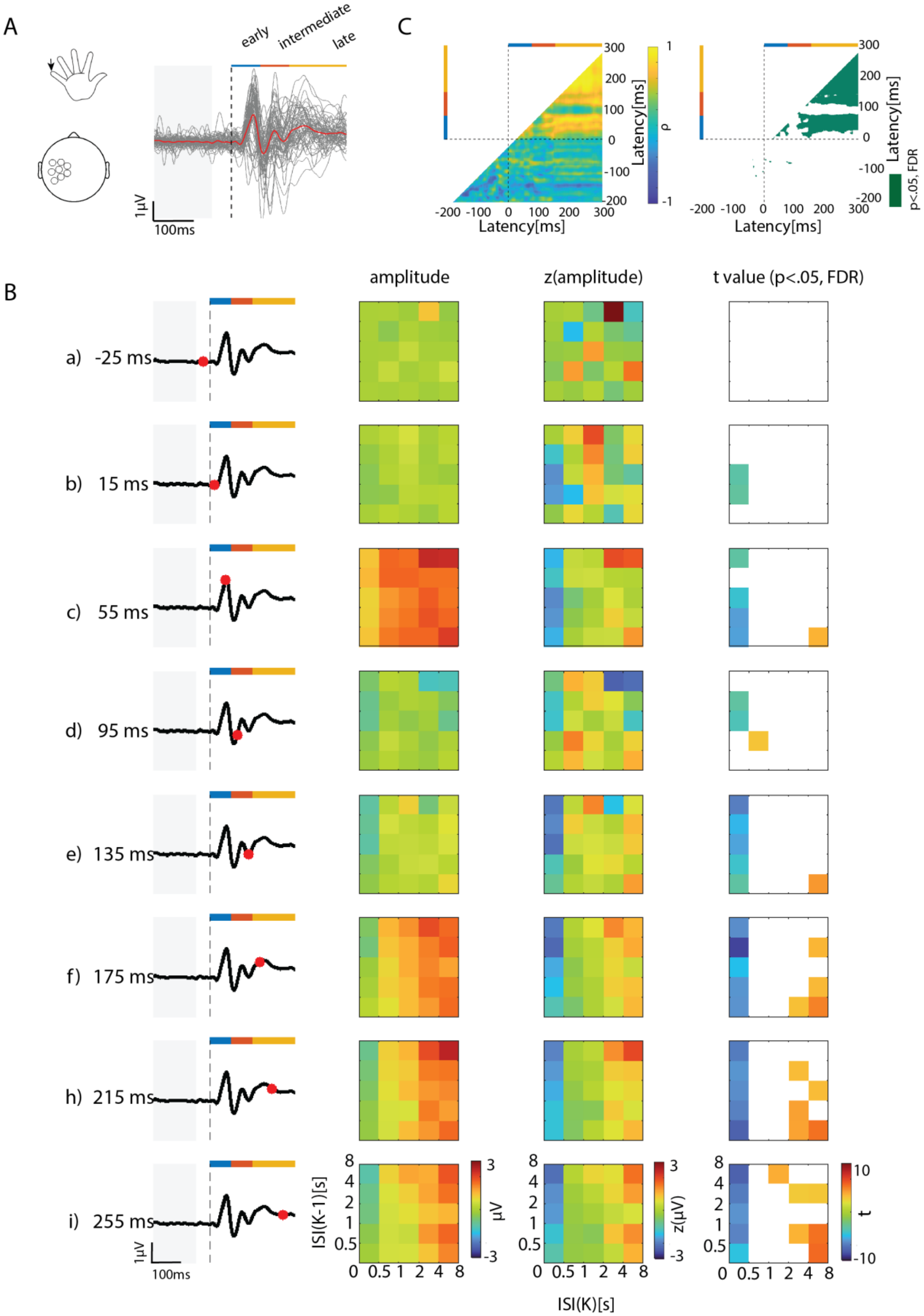

A
early
intermediate
late
1μV
100ms
C
300
200
100
0
-100
-200
-200 -100 0 100 200 300
Latency[ms]
ρ
1
-1
p<.05, FDR
B
amplitude
z(amplitude)
t value (p<.05, FDR)
a) -25 ms
b) 15 ms
c) 55 ms
d) 95 ms
e) 135 ms
f) 175 ms
h) 215 ms
i) 255 ms
ISI(K-1)[s]
8
4
2
1
0.5
0 0.5 1 2 4 8
μV
z(μV)
t
3
-3
10
-10
ISI(K)[s]

Supplementary Fig. S4 . The temporal dynamics of tactile inputs from index fingertip can shape the cortical processing at the electrode placed over the left somatosensory cortex. Please see the supplementary Fig. S2 for legend.

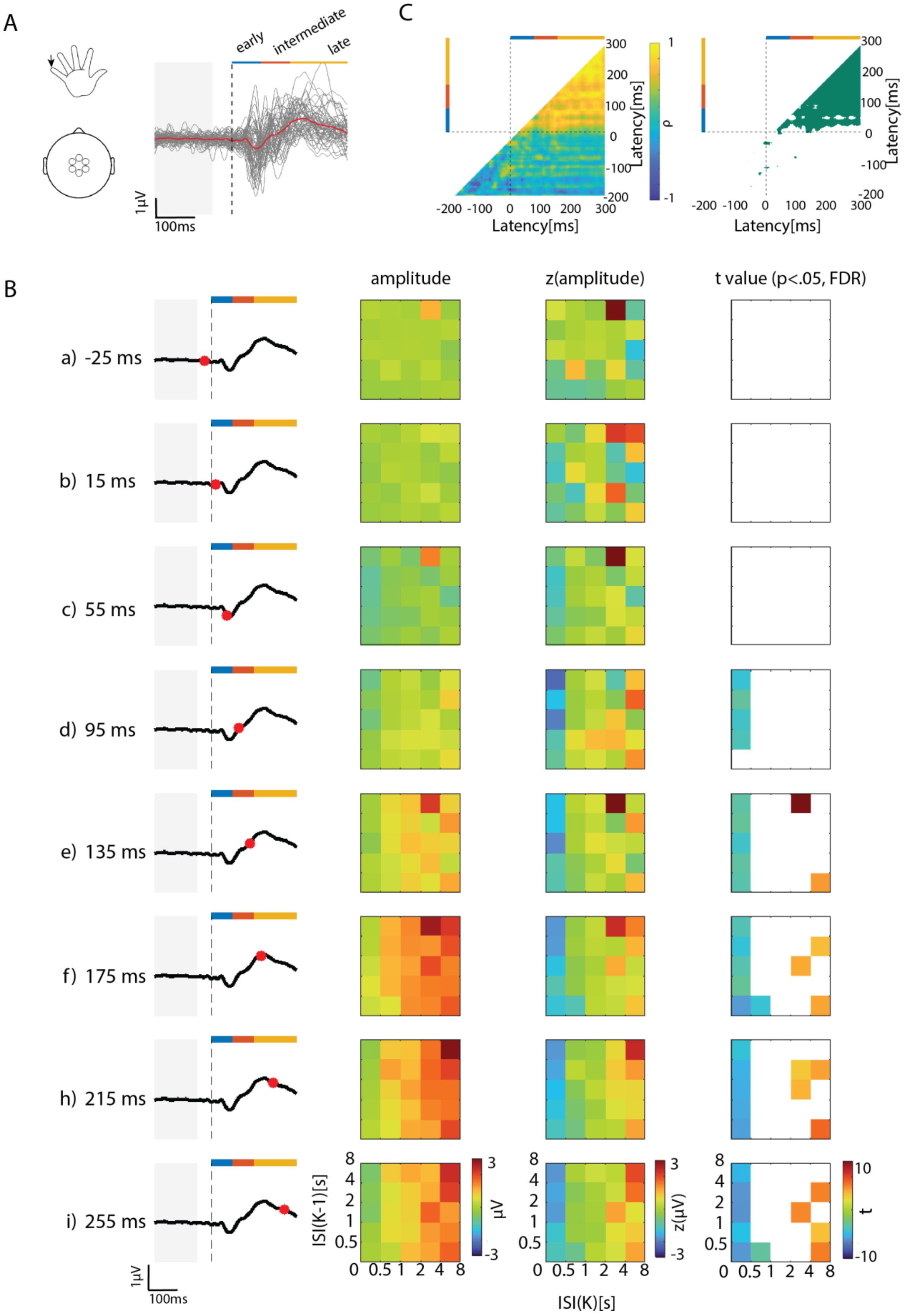
A
early
intermediate
late
1µV
100ms
C
Latency[ms]
ρ
B
amplitude
z(amplitude)
t value (p<.05, FDR)
a) -25 ms
b) 15 ms
c) 55 ms
d) 95 ms
e) 135 ms
f) 175 ms
h) 215 ms
i) 255 ms
ISI(K-1)[s]
µV
z(µV)
t
ISI(K)[s]

Supplementary Fig. S5. The temporal dynamics of tactile inputs from index fingertip can shape the cortical processing at the central electrode location. Please see the supplementary Fig. S1 for legend.

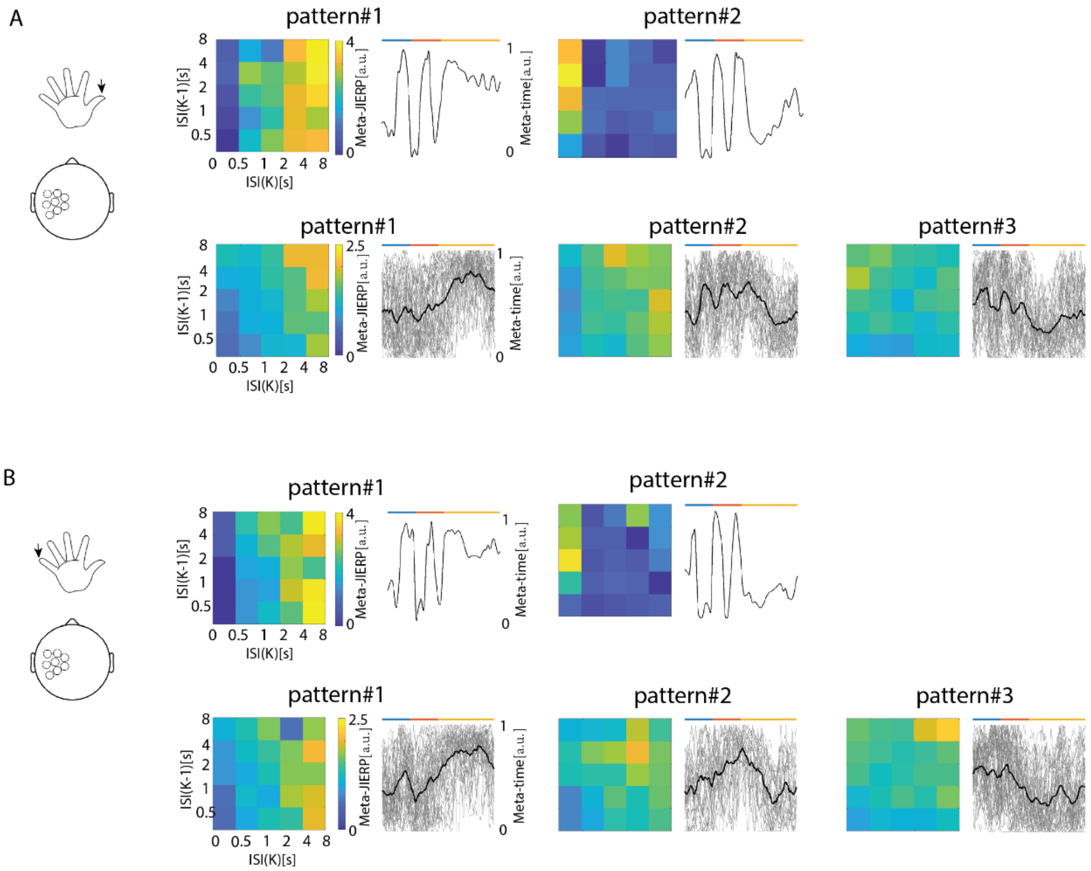

Supplementary Fig. S6. The typical JID-ERP patterns across different stages for other two fingers with the electrode placed over the left somatosensory cortex. (A) Typical *JID-ERP* patterns for thumb. The frequency for each electrode was shown as insert topography plot. The first row of right side showed the population level results based on the grand averaged EEG signals across the sample. The second row showed the individual level results after clustering all participants *meta-ERPs* and averaging *meta-JIDs* and *meta-ERPs* for each clusters. *Meta-ERPs* for each participant was shown as grey line and the averaged *meta-JIDs* was shown as dark line. Different scales were used for averaged *meta-JIDs* compared to (A). (B) Typical *JID-ERP* patterns for little finger. Same legend with (A).

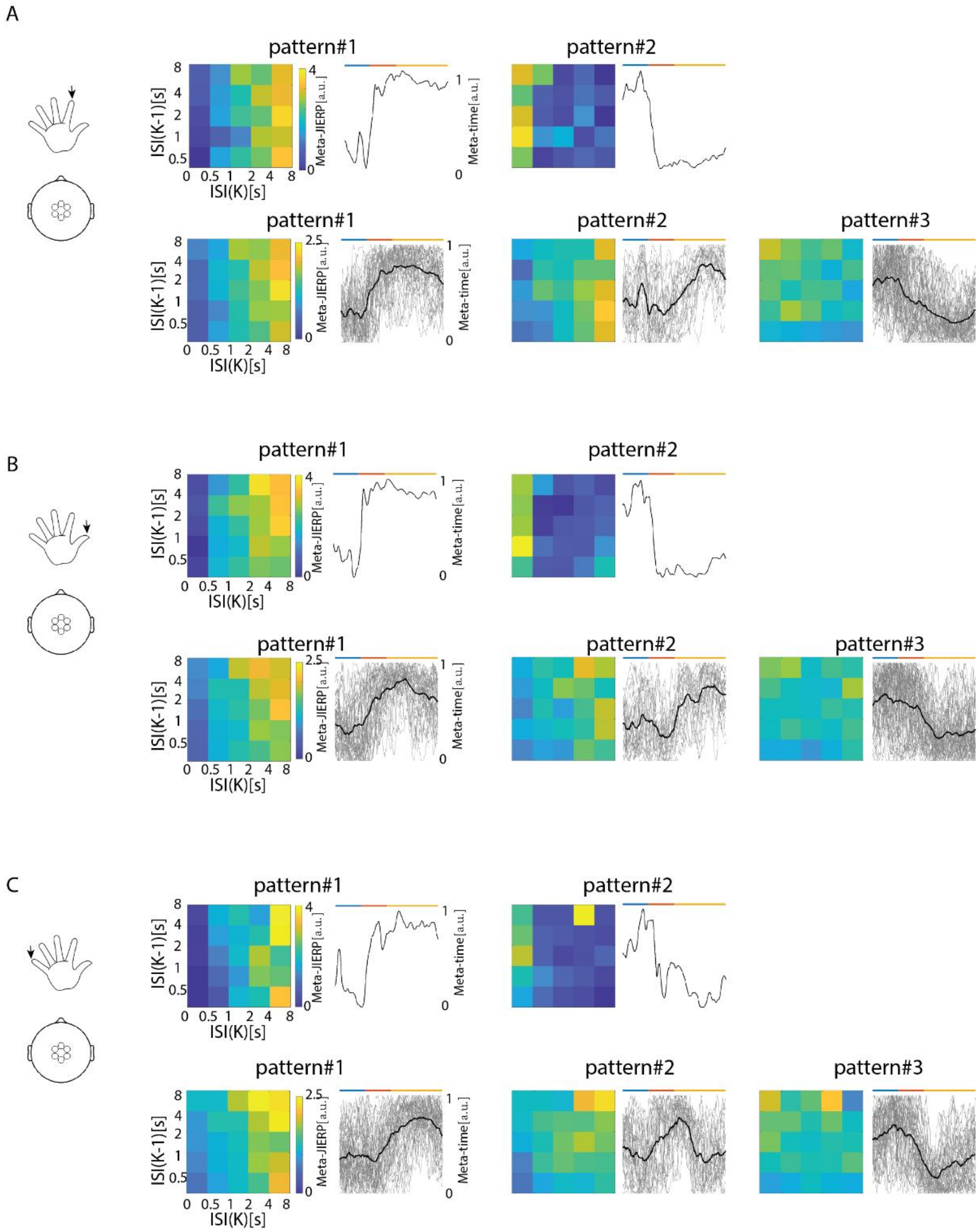

Supplementary Fig. S7. The typical JID-ERP patterns across different stages for all fingers with the electrode placed over the central electrode. (A) Typical JID-ERP patterns for index finger. (B) Typical JID-ERP patterns for thumb. (C) Typical JID-ERP patterns for little finger. Please see the supplementary Fig. S6 for the detailed legend.

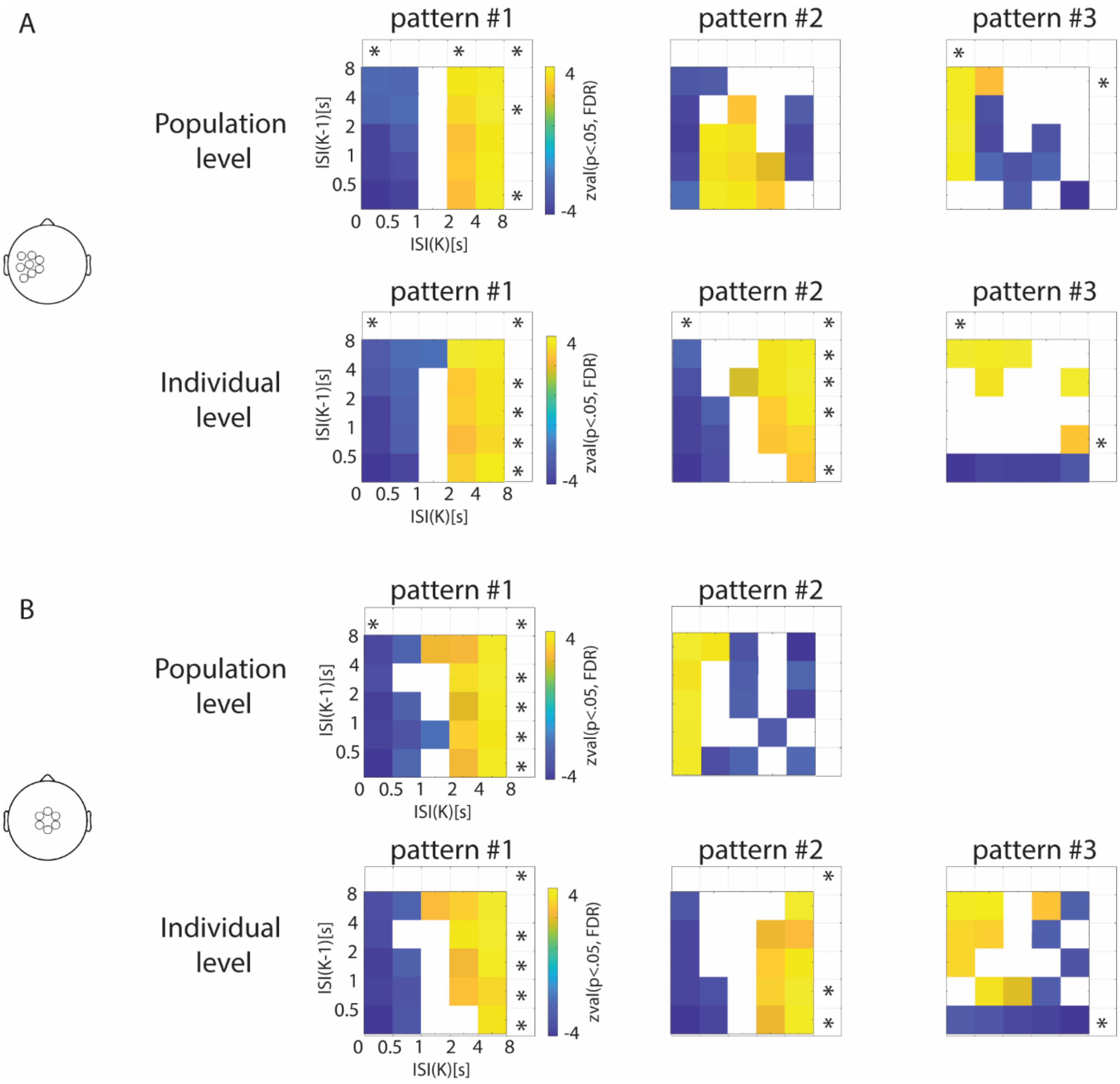

Supplementary Fig. S8. Statistical results of NNMF outputs for the index finger. Statistical results of one-sample Wilcoxon signed-rank tests (p < 0.05, FDR-corrected) examining whether each bin differs from the remaining bins, and Kendall's tau correlation tests (p < 0.05, FDR-corrected) examining whether gradient signal changes along the row (previous ISIs), column (penultimate ISIs), or diagonal (both previous and penultimate ISIs). (A) Left somatosensory electrode; (B) central electrode. The first row shows group-level prototypical JIERP patterns, and the second row shows individual-level results.

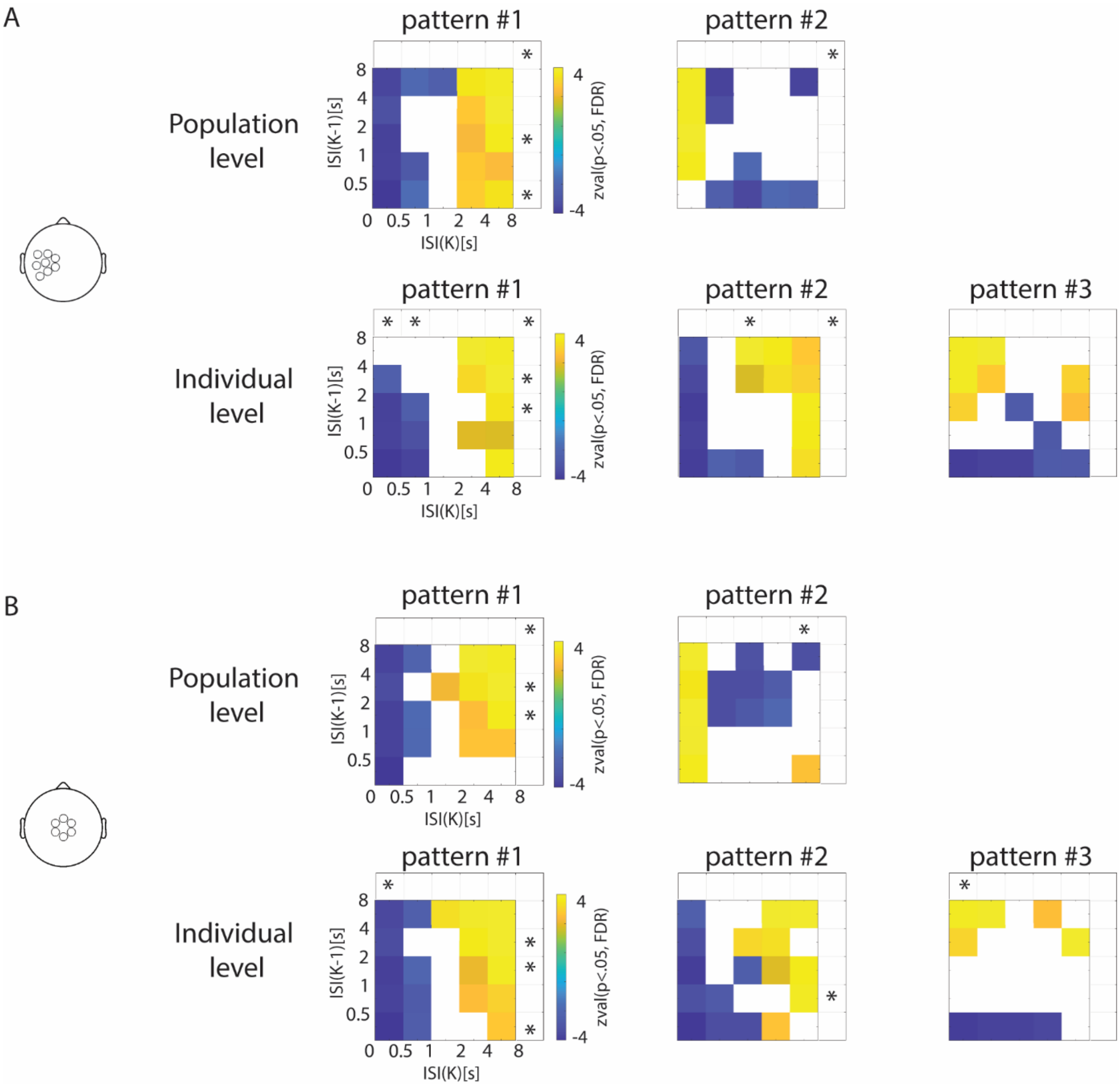

Supplementary Fig. S9. Statistical results of NNMF outputs for the thumb. Please see the supplementary Fig. S8 for the detailed legend.

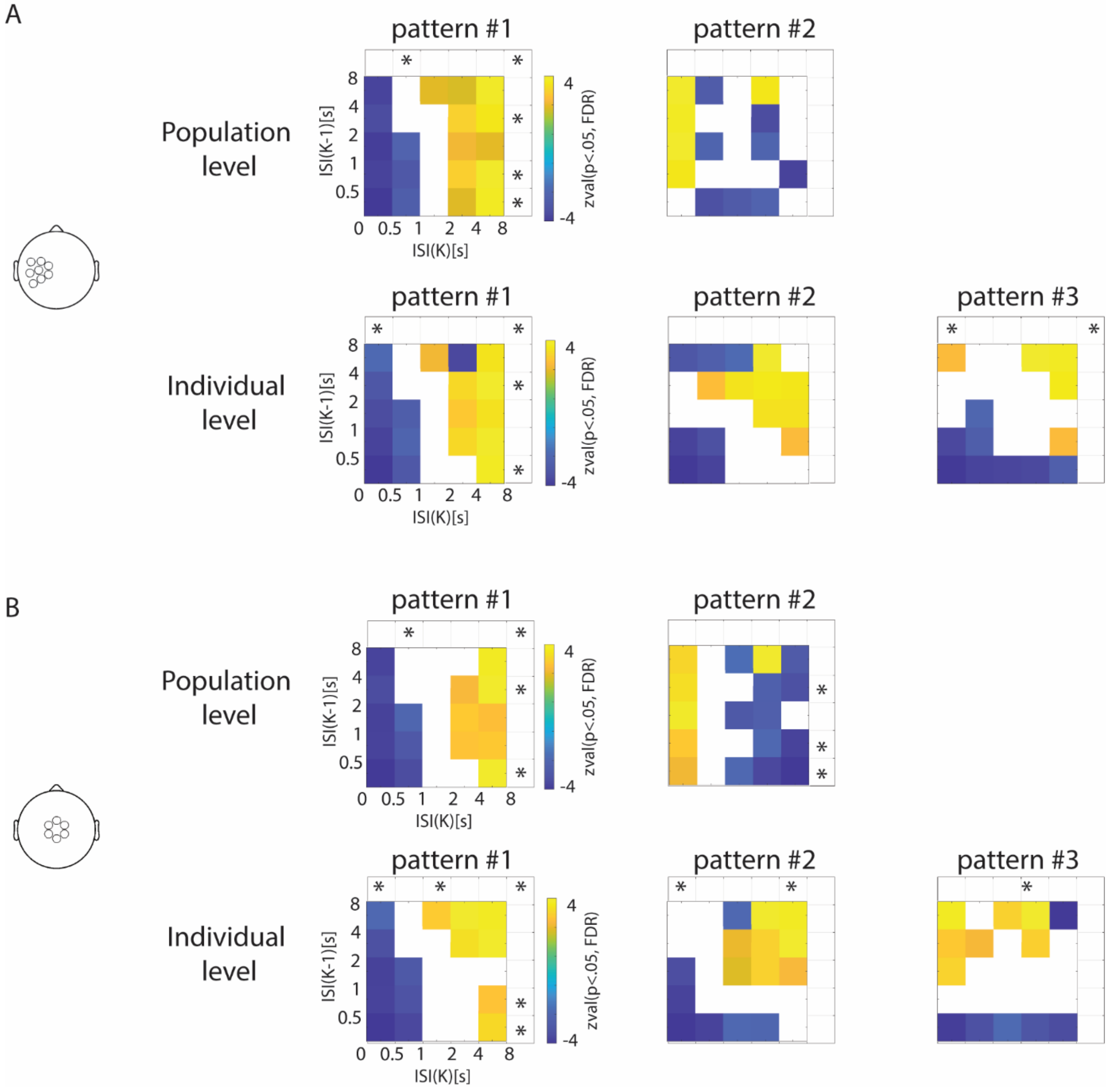

Supplementary Fig. S10. Statistical results of NNMF outputs for the little finger. Please see the supplementary Fig. S8 for the detailed legend.

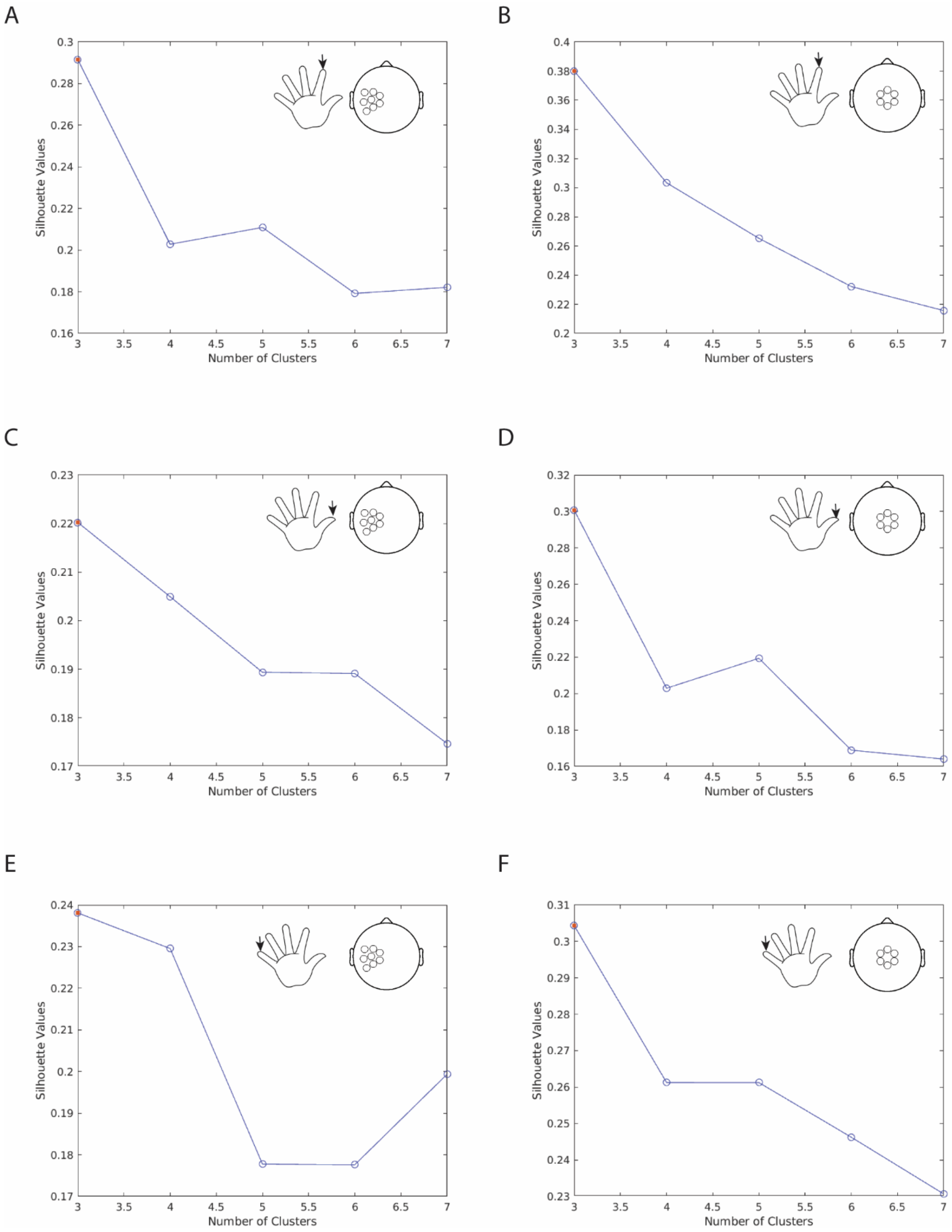

Supplementary Fig. S11. Optimal number of clusters (k, marked in red) determined by silhouette analysis. Silhouette-based clustering results for the gathered meta-times of the index finger at the left somatosensory electrode (A) and middle electrode (B); the thumb at the left somatosensory electrode (C) and middle electrode (D); and the middle finger at the left somatosensory electrode (E) and middle electrode (F).
